# Tissue maturation and development of mechanical properties in hyaluronic acid bioink-based cartilaginous constructs

**DOI:** 10.3389/fbioe.2026.1749259

**Published:** 2026-04-08

**Authors:** Paula Büttner, Jessica Faber, Jörg Teßmar, Philipp Stahlhut, Silvia Budday, Torsten Blunk

**Affiliations:** 1 Department of Trauma, Hand, Plastic and Reconstructive Surgery, University Hospital Würzburg, Würzburg, Germany; 2 Institute of Continuum Mechanics and Biomechanics, Friedrich-Alexander-Universität Erlangen-Nürnberg, Fürth, Germany; 3 Department of Functional Materials in Medicine and Dentistry and Bavarian Polymer Institute, University Hospital Würzburg, Würzburg, Germany

**Keywords:** bioengineering, biofabrication, biomechanics, cartilage, hyaluronic acid, extracellular matrix, chondrogenic differentiation

## Abstract

**Introduction:**

Bioprinting has emerged as a promising technology for cartilage tissue engineering. In cartilage, the mechanical properties are closely associated with the extracellular matrix (ECM) and of paramount importance for tissue functionality. However, to date mechanical analysis of biofabricated cartilaginous constructs is often limited to single parameters, and a detailed analysis of the concomitant development of ECM and mechanical properties is lacking. Therefore, in this study, we investigate the post-fabrication development of the ECM and comprehensively analyze the large-strain viscoelastic properties of cartilaginous constructs made from mesenchymal stromal cells (MSC) in a hyaluronic acid (HA)-based bioink.

**Methods:**

MSC are embedded in a HA-based bioink and chondrogenically differentiated post-production for 6 weeks. A time-matched analysis is performed monitoring the ECM development represented by glycosaminoglycans and collagen as well as the changes in mechanical properties, such as hysteresis, classical shear modulus, nonlinearity and stress relaxation, using a multimodal testing approach at six different time points.

**Results:**

Distinctly increasing amounts of ECM components are detected in the cartilaginous constructs over the whole cultivation period. Glycosaminoglycans and collagen content increase by 57- and 52-fold, respectively, from day 1 to day 42. In turn, the ECM development markedly influences the overall multimodal mechanical response over time, leading to distinct changes in nonlinear and stress relaxation behavior and a 265-fold increase in hysteresis and a 174-fold increase in classical shear modulus reaching 50 kPa in total. We find a strong correlation between the amount of glycosaminoglycans and collagen, and the classical shear modulus as well as the hysteresis during cyclic loading. When partially inhibiting collagen production in the constructs, the robustness of the correlation between collagen and classical shear modulus is confirmed.

**Discussion:**

Our study underlines the substantial impact of matrix components on the mechanical behavior in biofabricated chondrogenic constructs. The results emphasize the importance of a comprehensive analysis of the mechanical properties. We propose a matrix-based prediction function that can estimate the classical shear modulus of constructs made from HA-based bioinks. Overall, our findings contribute to the understanding of tissue maturation in engineered constructs for cartilage regeneration.

## 1 Introduction

Articular cartilage is an essential tissue for joint movement, transferring loads from the joint to the underlying bone. While healthy cartilage provides the necessary mechanical stability, damaged cartilage, e.g., due to injury or diseases such as osteoarthritis (OA), shows alterations in the composition of the extracellular matrix (ECM), leading to softening in mechanical properties and matrix degradation, which eventually causes painful joint movement for patients (Sophia Fox et al., 2009; Pueyo Moliner et al., 2025). Healthy articular cartilage is a poro-viscoelastic material, consisting of a fluid phase due to its high water content and a solid phase, containing ECM components such as proteoglycans and collagens (Mow and Guo, 2002; Little et al., 2011; Eschweiler et al., 2021). The mechanical response of cartilage is characterized by a highly nonlinear, hysteretic behavior with substantial conditioning between the first and second cycle during cyclic loading and a pronounced stress relaxation behavior in compression and tension (June et al., 2009; Edelsten et al., 2010; Tanaka et al., 2014; Weizel et al., 2020; Han et al., 2021; Krakowski et al., 2024). Current clinical approaches to treat damaged cartilage, ranging from drug-based treatment to surgical approaches, often lack sufficient long-term success (Richter et al., 2016; Song and Park, 2019; Kloppenburg and Berenbaum, 2020; D'Arcy et al., 2021). Other treatment techniques are based on tissue engineering (TE) (Makris et al., 2015; Peng et al., 2025). TE-based therapies, however, can lead to formation of fibrocartilage with mechanical properties different from original hyaline cartilage (Li et al., 2025).

Biofabrication including 3D bioprinting has emerged as a promising technology for engineering 3D tissues, potentially allowing the precise control of cell density, ECM composition, and mechanical properties of the final construct (Levato et al., 2020). Various biofabrication approaches have been established in the last decade to produce cartilage constructs, utilizing various polymers (synthetic or natural) and cell sources (primary chondrocytes, cartilage progenitor cells, mesenchymal stromal cells (MSC), induced pluripotent stem cells) to either develop stand-alone bioinks or to combine them with supporting structures. Bioinks can be combined with rapidly removable supporting structures, e.g., made from pluronic (Levato et al., 2017), to improve the printability, or long-term degradable support structures, e.g., scaffolds made from polycaprolactone (PCL), which can increase the overall stability and stiffness of the printed construct (Hauptstein et al., 2020; Spauwen et al., 2025). Depending on the construct type, physiological loading of the sample can positively influence the ECM production and chondrocyte migration (De Croos et al., 2006; Nicodemus and Bryant, 2010; Musumeci, 2016; Vaca-Gonzalez et al., 2019; Gilbert et al., 2021; Goldberg-Bockhorn et al., 2022; Jia et al., 2023). However, if an integrated scaffold is too rigid, it prevents transfer of the mechanical loading due to stress shielding (Spauwen et al., 2025). In turn, stand-alone bioinks that allow for the production and distribution of ECM throughout the constructs can enable cartilaginous tissue maturation without the influence of a scaffold. However, detailed time-matched post-fabrication analyses of ECM components produced by the cells and the corresponding mechanical properties of the biofabricated construct are lacking.

For the detailed mechanical analysis, various measuring techniques can be used *in vitro*, e.g., based on tensile, compressive and shear testing techniques. The majority of studies performing mechanical analysis are based on compression testing (Patel et al., 2019). Compressive forces can be applied either to the whole construct in a confined or unconfined measurement or via indentation (Mow et al., 1989; Korhonen et al., 2002). Most studies on cartilage surrogates are restricted to the determination of the Young’s modulus, calculated by the slope of the stress and strain curve in the linear elastic region (Patel et al., 2019; Welsh and Sikder, 2025). However, since the Young’s modulus only holds for small deformations and for an individual loading condition, more complex constitutive models are necessary to fully characterize the highly complex mechanical response of cartilage undergoing large deformations as those occurring *in vivo* (Weizel et al., 2022).

In a previous study, we have established a hyaluronic acid (HA)-based bioink platform, allowing the precise control of network density by varying the polymer chain lengths and polymer content while maintaining good printability (Hauptstein et al., 2022). Bioinks with low polymer content yielding a low crosslinking density provided favorable properties for the homogeneous distribution of ECM components during chondrogenic differentiation of MSC, in turn resulting in an increase in construct stiffness (fold change >70). However, the Young’s modulus as a measure of stiffness was the only mechanical parameter determined, and only an endpoint analysis of ECM development and stiffness increase after 3 weeks was performed (Hauptstein et al., 2022).

In the present study, we focus on a detailed biological and mechanical post-fabrication analysis of cartilaginous constructs made from a stand-alone HA-based bioink. Chondrogenic differentiation of MSC was analyzed regarding ECM production at several time points over a six-week period. Concomitantly, a comprehensive time-matched large-strain multimodal analysis evaluating several mechanical properties such as the classical shear modulus, hysteresis, nonlinearity, conditioning as well as the stress relaxation behavior was performed in the chondrogenic constructs. Furthermore, we evaluated the contributions of matrix components to the macroscopic mechanical response and present a matrix-based prediction function that can estimate the classical shear modulus of the chondrogenic constructs.

## 2 Materials and methods

### 2.1 2D cell culture

Isolation of mesenchymal stromal cells (MSC) was performed as previously described in (Stichler et al., 2017), with informed consent of all patients and approval of the local ethics committee of the University of Würzburg (186/18). Cells were expanded in 2D culture at 37 °C and 5% CO_2_ in Dulbecco’s Modified Eagle’s medium/Ham’s F12 (DMEM/F12) (11320-074, Gibco, Waltham, MA, United States), supplemented with 10% fetal calf serum (FCS) (10270-106, Gibco), 1% penicillin/streptomycin (P/S) (15140-122, Gibco) and 3 ng/mL basic fibroblast growth factor (bFGF) (571506, Biolegend, San Diego, CA, United States).

### 2.2 Hydrogel preparation and 3D cell culture

HA-SH hydrogels were prepared as previously described (Hauptstein et al., 2022), with 0.5% of a 538 kDa thiolated HA (HA-SH, 41% degree of substitution), 0.5% 6 kDa PEG-diacryl and 1% 6 kDa PEG-diallyl using lithium-phenyl-2,4,6-trimethylbenzoylphosphinate (LAP) (900889, Sigma Aldrich, St. Louis, MA, United States) as photoinitiator. MSC were harvested (passage 3, 20*10^6^ cells per ml hydrogel), mixed with the hydrogel and transferred to a printing cartridge (7012074, Nordson EFD, Westlake, OH, United States), pre-crosslinked for 2 h at 37 °C, and afterwards extruded through a 23G nozzle (7018302, Nordson EFD) with a printing pressure of 50–100 kPa. To ensure uniform constructs for mechanical analysis, all constructs were cast into 40 µL cylindrical molds (*d* = 5 mm, *h* = 2 mm) after the printing process. All samples were finally crosslinked at 405 nm for 2 min (1,500 mJ/cm^2^). Constructs were incubated over a period of 42 days at 37 °C and 5% CO_2_ in chondrogenic differentiation medium (DMEM high glucose medium (D5796, Sigma Aldrich) supplemented with 1% ITS + premix (354352, Corning, Corning, NY, United States) 1% P/S, 0.1 µM dexamethasone (50-02-2, Sigma Aldrich), 50 μg/ml L-ascorbic acid 2-phosphate sesquimagnesium salt hydrate (A8960, Sigma Aldrich), 40 μg/mL L-proline (147-85-3, Sigma Aldrich) and 10 ng/mL TGF-*β*1(CA59, Bon-Opus Bioscience, Millburn, NJ, United States)). Hydrogels were harvested on day 1, 3, 7, 14, 21 and 42 for further analysis.

### 2.3 Cell viability assay

Cell viability of the cylindric 3D samples was analyzed using live/dead staining on day 1, 3, 7, 14, 21, and 42. 3D constructs were washed with phosphate-buffered saline (PBS) (14190-250, Gibco) and bisected prior to staining. The staining solution (calcein-acetoxymethylester and ethidium homodimer III, BOT-30002-T, Biotium, Fremont, CA, United States) was then added and the samples were incubated for 45 min at room temperature. Images of the construct cross sections were taken using an Olympus BX51 microscope (Olympus, Hamburg, Germany).

### 2.4 Printability assay

Printability was analyzed using the intersection diagonal of printed grid structures. The HA-SH-based bioink was pre-crosslinked at 37 °C. Afterwards, six grid structures were printed during a 30-min time period using a 23G nozzle. Images of the printed grid structures were acquired with a stereo microscope (SteREO Discovery. V20, Carl Zeiss, Jena, Germany). Images were further analyzed using NIH ImageJ Fiji software (version 1.54f), wherefore the intersection diagonal of 8 grids per condition were measured. For the analysis, the mean of the mean (Equation 1)

$$mean\;\;of\;mean=\frac{\sum mean\;\;of\;intersection\;diagonal}{number\;of\;grids}$$ (1)

was calculated using OriginLab (OriginLab Corporation, MA, United States) software. Viability of embedded cells in printed grid structure was assessed by incubating the grids in staining solution (see Section 2.3) for 45 min. Overview images were then taken using an Olympus BX51 microscope (Olympus).

### 2.5 Microstructural analysis using scanning electron microscopy (Cryo-SEM)

HA-SH hydrogel samples with cells were prepared as described above with 20*10^6^ cells per ml bioink and either stored in chondrogenic differentiation medium overnight (O/N) or cultivated for 3 weeks. HA-SH hydrogels without cells were prepared and stored accordingly. Samples were, if applicable, washed with PBS to remove cell culture medium and afterwards fixed in a sandwich of 3 mm aluminum plates in a 2 mm notch. For rapid freezing, slushed nitrogen at −210 °C was used. An EM VCT100 cryo-shuttle (Leica Microsystems, Wetzlar, Germany) under vacuum (<1 × 10^3^ mbar) and cooled with liquid nitrogen was used to transport samples between steps. One aluminum plate was knocked off to generate a freshly fractured hydrogel surface and samples were freeze edged in an ACE400 sputter coater machine (Leica Microsystems) under high vacuum (<1 × 10^3^ mbar) for 15 min at −85 °C. Afterwards, samples were sputtered with 2.5 nm platinum before placing them into the SEM chamber (Crossbeam 340, Zeiss). Imaging was performed at −156 °C at an acceleration voltage of 8 kV.

Microstructure of the gels was further analyzed using the NIH ImageJ Fiji software (version 1.54f). For this purpose, three regions of interest (ROI) per image of each group were analyzed, resulting in six analyzed ROIs for each group. Pores were marked adjusting the threshold of the images and afterwards analyzed using the Analyze Particles Function of ImageJ. Statistical analysis was performed with the Mann-Whitney test, using the OriginLab (OriginLab Corporation, MA, United States) software.

### 2.6 Histological and immunohistochemical analysis

For histological and immunohistochemical analysis, the constructs were bisected, fixed with 4% formalin solution (9713.1000, VWR by Avantor, Radnor, PA, United States), subsequently embedded in Tissue Tek O.C.T (4583, Sakura Finetek, Torrance, CA, United States) and stored in a wet chamber at room temperature overnight. The samples were then shock frozen with aligned cutting edge in liquid nitrogen and stored in −20 °C until further processing. The samples were sliced (8 µm per longitudinal section) using a cryostat (CM, 3050S, Leica, Wetzlar, Germany) and mounted on Superfrost Plus ™ Microscope Slides (631-0108, VWR by Avantor). For total collagen staining, the samples were stained for 1 h with 0.1% picrosirius red (0.1% direct red 80, 365548-5G, Sigma Aldrich, in 1.2% picric acid, A2520, AppliChem, Darmstadt, Germany). For counterstaining of the nuclei, the samples were stained with Mayer’s hemalum (1.09249.2500, Sigma Aldrich). For staining of glycosaminoglycans (GAG), the samples were stained using 0.1% safranin O (S2255-25G, Sigma Aldrich) and counterstained with 0.02% fast green (F7252, Sigma Aldrich) and Mayer’s hemalum. Histological images were acquired with Olympus IX51 microscope (Olympus). For immunohistochemistry, the samples were digested with Proteinase K (S3020, Dako, Agilent Technologies, Santa Clara, CA, United States) and subsequently incubated O/N with the following primary antibodies: anti-aggrecan (1:300; 969D4D11, Thermo Scientific, Waltham, MA, United States); anti-collagen type II (1:200, CP18, Sigma Aldrich); anti-collagen type X (1:50, 14-9771-82, Invitrogen, Waltham, MA, United States), anti-collagen type I (1:200; ab34710, Abcam, Cambridge, United Kingdom). As secondary antibodies, goat anti-mouse Alexa488 (1:300, 115-545-146, Jackson ImmunoResearch, West Grove, PA, United States) or goat anti-rabbit Alexa488 (1:300; ab15007, Abcam) were used. All antibodies were diluted in 1% BSA/PBS (A9418, Sigma Aldrich). All samples were mounted with DAPI mounting medium (SCR-038448, dianova, Hamburg, Germany) and imaged with Olympus BX51 microscope (Olympus). All images were processed using cellSens Software (Olympus).

### 2.7 Biochemical analysis

For the quantification of ECM components, the constructs were harvested at all analyzed timepoints (day 1, 3, 7, 14, 21, and 42) and homogenized in a PBE-cysteine buffer (E9884/30412/C7352, Sigma Aldrich) for 5 min at 25 Hz using a Tissue Lyser (MM400, Retsch, Haan, Germany). Additionally, all samples were digested for 20 h with 3 U/mL Papain (LS003126, Worthington, NJ, United States) at 60 °C. The DNA content was quantified using Hoechst 33258 (09460, Polysciences, Hirschberg, Germany) and a standard curve based on salmon sperm DNA (31149, Sigma Aldrich) (Kim et al., 1988). Samples were analyzed with a spectrofluorometer at 340 and 465 nm. The GAG content was quantified using dimethylmethylene blue (DMMB) (341088, Sigma Aldrich) staining solution enabling a measurement at 525 nm (Farndale et al., 1986) and using chondroitin sulfate (C9819, Sigma Aldrich) as standard. Collagen content was analyzed after an acid hydrolysis with a hydroxyproline assay (Woessner, 1961), using hydroxyproline (1.04506, Merck, Darmstadt, Germany) as standard. All assays were performed using an Infinite M200 Pro (Tecan, Crailsheim, Germany). The concentration per well was determined using triplicates of each condition and calculated as follows setting x as sample concentration, y as absorbance of the sample/blank, *c* as y-intercept of the standard curve and *m* as slope of the standard curve (Equation 2):

$$x=\frac{\left(y_{sample}-y_{blank}\right)-c}{m}$$ (2)

### 2.8 Mechanical characterization

Mechanical characterization was performed at all time points, see Section 2.2. To perform multimodal mechanical tests in compression and tension, a Discovery Hybrid Rheometer HR 30 from TA instruments (New Castle, DE, United States) was used. After calibration of the rheometer, a 6 mm circular piece of fine sandpaper with a grain size of 180 μm was glued to the upper and lower specimen holder to improve the adhesion between the sample and the smooth specimen holders. A stainless steel plate with *d* = 8 mm for the upper and an anodized aluminum plate with *d* = 40 mm for the lower specimen holder was used. Afterwards, a thin layer of cyanoacrylate adhesive Pattex superglue ultra gel (Henkel AG & Co. KGaA, Düsseldorf, Germany) was added to the upper specimen holder; the sample was placed on a spatula and glued in the center of the upper geometry. A thin layer of the same adhesive was added to the lower specimen holder. The upper specimen holder was lowered until a full contact between the sample and the lower specimen holder was visually confirmed. After attachment, the mean diameter of each sample was determined by using the ImageJ Fiji software (version 1.54p). A transparent immersion cup allowed us to easily track the contact before and during testing. After a waiting time of 90 s for the adhesive to dry, samples were immersed in differentiation medium to ensure hydration during testing. Cylindrical samples were tested *in vitro* under loading conditions representative of those experienced by implants *in vivo*. The testing procedure established in our previous study on human articular cartilage (Faber et al., 2025a) formed the basis for the experiments conducted in this study. To mimic physiological loading conditions at moderate strain rates for walking (Li and Herzog, 2004; Carter et al., 2015; Currey et al., 2019; Komeili et al., 2019; Patel et al., 2019), cyclic loading experiments in compression up to 20% strain and in tension up to 2.5% strain were conducted at a loading rate of 40 μm/s (average strain rates: 1.7%/s for control group, 2.1%/s for P4H-inhibited group, 1.5%/s for cell-free constructs) with a total of three loading cycles. Subsequently, stress relaxation experiments in compression up to the same maximum strain of 20% were performed at a 2.5 times higher loading rate of 100 μm/s (average strain rates: 4.3%/s for control group, 5.2%/s for P4H-inhibited group, 3.8%/s for cell-free constructs) and a holding time of 300s (Table 1). All tests were performed at 37 °C.

**TABLE 1:** Multimodal testing protocol for chondrogenic constructs.

Experimental tests	Loading mode	Stretch	Loading rate in µm/s	Holding time in s
Cyclic loading	Compression and tension	0.8 and 1.025	40	​
Stress relaxation	Compression	0.8	100	300

### 2.9 Hyperelastic constitutive modeling

To characterize the deformation of the samples during testing, we used the nonlinear equations of continuum mechanics and introduced the deformation map ***φ*** (***X***,*t*), which maps tissue from the undeformed configuration to the deformed configuration. The deformation gradient ***F*** (***X***,*t*) = ∇_**X**_ *φ*(***X***,*t*) maps line elements from the undeformed configuration to the corresponding line elements in the deformed configuration, where ***X*** and ***x*** denote the corresponding position vectors, respectively. The principal stretches $\lambda_{i},i=1,2,3$ , are defined as the square roots of the eigenvalues of the left and right Cauchy Green strain tensors, given by ***b*** = ***FF*** ^T^ and ***C*** = ***F*** ^T^ ***F***, where the deformation gradient is [***F***] = diag $\left\{\lambda_{1},\lambda_{2},\lambda_{3}\right\}$ .

To characterize the constitutive behavior of a hyperelastic material, we split the strain-energy function $\psi^{O}$ into an isochoric $\psi_{iso}^{O}$ and a volumetric contribution $\psi_{vol}^{O}$ . For the isochoric contribution, we consider the modified one-term Ogden model (Equation 3) and the two-term Ogden model (Equation 4) (Ogden, 1972) with its phenomenological, isotropic hyperelastic strain-energy functions

$$\psi_{\text{iso}}^{1T\;\text{Ogden}}=\frac{2\mu }{{\alpha_{1}}^{2}}\left[{\bar{\lambda }}_{1}^{\alpha_{1}}+{\bar{\lambda }}_{2}^{\alpha_{1}}+{\bar{\lambda }}_{3}^{\alpha_{1}}-3\right]$$ (3)

$$\psi_{\text{iso}}^{2T\;\text{Ogden}}=\frac{{µ}_{1}}{\alpha_{1}}\left[{\bar{\lambda }}_{1}^{\alpha_{1}}+{\bar{\lambda }}_{2}^{\alpha_{1}}+{\bar{\lambda }}_{3}^{\alpha_{1}}-3\right]+\frac{{µ}_{2}}{\alpha_{2}}\left[{\bar{\lambda }}_{1}^{\alpha_{2}}+{\bar{\lambda }}_{2}^{\alpha_{2}}+{\bar{\lambda }}_{3}^{\alpha_{2}}-3\right]$$ (4)

The modified one-term Ogden model is formulated in terms of the classical shear modulus µ , the nonlinearity parameter $\alpha_{1}$ , which are related through

$$\mu =\sum_{i=1}^{n}\frac{\mu_{i}\alpha_{i}}{2}=\frac{\mu_{1}\alpha_{1}}{2}$$ (5)

known from the linear theory (Equation 5), and the isochoric principal stretches ${\bar{\lambda }}_{1},{\bar{\lambda }}_{2},{\bar{\lambda }}_{3}$ (Holzapfel, 2002).

The two-term Ogden model is formulated in terms of two shear moduli *μ*_1_ and *μ*_2,_ two nonlinearity parameters $\alpha_{1}$ and $\alpha_{2}$ , and the isochoric principal stretches ${\bar{\lambda }}_{1},{\bar{\lambda }}_{2},{\bar{\lambda }}_{3}$ . In the linear regime, the relation

$$\mu =\sum_{i=1}^{n}\frac{\mu_{i}\alpha_{i}}{2}=\frac{\mu_{1}\alpha_{1}}{2}+\frac{\mu_{2}\alpha_{2}}{2}$$ (6)

holds (Holzapfel, 2002) (Equation 6).

For the volumetric contribution, we choose the generalized strain-energy function from Ogden (Ogden, 1972) with an empirical coefficient of −2 (Simo and Miehe, 1992; Holzapfel, 2002) (Equation 7)

$$\psi_{\text{vol}}^{O}=\frac{\kappa }{4}\left(-2\ln J+J^{2}-1\right)$$ (7)

where κ is the bulk modulus, which is defined as

$$\kappa =\frac{2\mu \left(1+\nu \right)}{3\left(1-2\nu \right)}$$ (8)

in terms of the Poisson’s ratio in the linear elastic regime (Equation 8). As it is common for hydrogels (Distler et al., 2021; Faber et al., 2025b), we assumed incompressibility of HA-SH hydrogels due to their high water content and set ν=0.49 (near incompressibility) to avoid numerical problems. In the linear regime, the (apparent) Young’s modulus (Equation 9) can be calculated as a function of the classical shear modulus *µ* and the Poisson’s ratio *ν* to

$$E_{\text{app}}=2\mu \left(1+\nu \right)\approx 3µ.$$ (9)

### 2.10 Data preprocessing

We used the data of the unconditioned and conditioned material responses during the first and third cycle of cyclic loading in both uniaxial compression and uniaxial tension and averaged the loading and unloading curves to approximate the hyperelastic, time-independent material responses. As both loading modes contributed with the same number of data points to the residual vector, we introduced an explicit weighting mechanism into the cost function *χ*2 by weighting compression with a ratio of 8:1 compared to tension loading. For further details regarding the data preprocessing, we refer to our previous studies (Hinrichsen et al., 2023; Faber et al., 2025b).

### 2.11 Inverse parameter identification

We coupled an inverse parameter identification scheme with finite element simulations to capture the actual boundary conditions including inhomogeneous deformations of the glued samples during testing to identify the optimal set of material parameters (Faber et al., 2022; Hinrichsen et al., 2023). The boundary constraints for the modified one- and two-term Ogden model were defined in Table 2. For further details regarding the parameter identification scheme, we refer to our previous studies (Hinrichsen et al., 2023; Faber et al., 2025b).

**TABLE 2:** Boundary constraints used during the optimization of the finite element simulations to determine the hyperelastic material parameters for the modified one- and two-term Ogden model.

One-term Ogden		Two-term Ogden			
*α*_1_	*µ* in Pa	*α*_1_	*α*_2_	*µ*_1_ in Pa	*µ*_2_ in Pa
( −∞,+∞ )	(0, + ∞ )	(- ∞ , −1)	(1, + ∞ )	(- ∞ , −1)	(1, + ∞ )

### 2.12 Statistical analysis

Normal distribution of the data was tested using Kolmogorov-Smirnov test. Further statistical analysis for pore size analysis was performed using Mann-Whitney test. Biochemical quantification and mechanical experiments of constructs were analyzed by one-way ANOVA with Bonferroni *post hoc* test using Origin Pro 2023 software. A *p*-value lower than 0.05 was considered to be significant (*p < 0.05). To evaluate the capability of the one- and two-term Ogden models to capture the time-independent response of HA-SH hydrogels, the quality of the fitting results was quantified in terms of the coefficient of determination (*R* ^2^) between experimental data and model output for compression and tension.

## 3 Results

### 3.1 ECM development

#### 3.1.1 Printability and microstructural changes of the bioink

**FIGURE 1:**
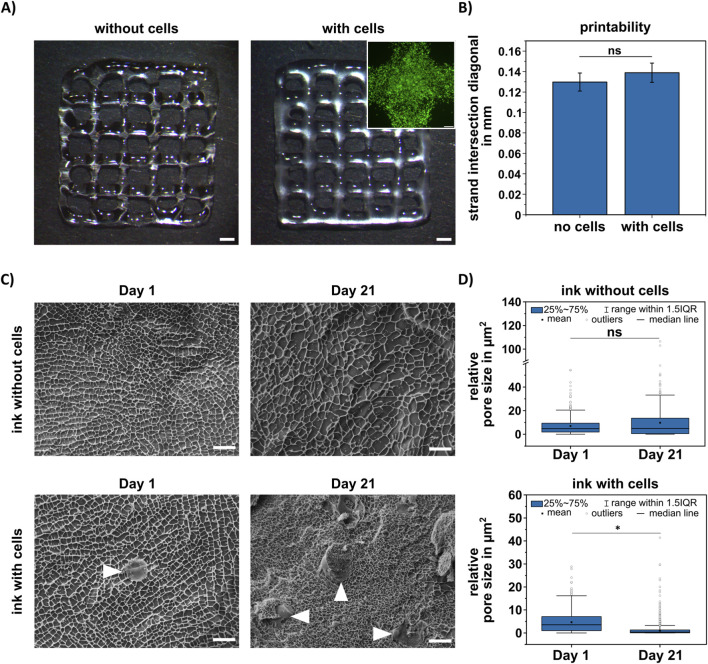
Printability and microstructure analysis of HA-SH-based bioink. **(A)** 3D printed grid structures without (left) and with embedded cells (right), scale bar represents 1 mm. Upper right image: live/dead staining of embedded MSC in printed intersection, scale bar represents 200 µm. **(B)** Quantification of intersection diagonal of printed strands (n = 8). Data are presented as mean ± standard deviation, statistically significant differences are indicated by * (p < 0.05), ns = no significant difference. **(C)** Microstructure visualization using cryo-SEM images of hydrogels without (top) and with (bottom) embedded cells, white arrows indicating position of embedded cells, scale bar represents 10 µm. **(D)** Relative pore size analysis of cryo-SEM images. Data are presented as median with 25 and 75 percentiles. Please note: The measured pore size represents the relative, not the actual pore size, due to the freezing process.

For the post-fabrication analysis of chondrogenic constructs, a previously established HA-based bioink was utilized (Hauptstein et al., 2022). For this study, we used a 538 kDa HA-SH with a 41% degree of substitution; the final total polymer content remained at 2%. Printability without and with embedded cells was demonstrated to be similar, while maintaining a high cell viability (Figure 1A). Quantification of the strand intersectional diagonals showed no significant difference between the bioink without and with embedded cells (Figure 1B). The microstructure of the bioink without and with embedded cells was additionally analyzed within 24 h after preparation as well as after 21 days using cryo-scanning electron microscopy (cryo-SEM) (Figure 1C). The constructs without cells showed no significant changes in pore size over time, while for the constructs with embedded cells, a decrease in pore size over time was observed (Figure 1D), likely due to the development of ECM.

#### 3.1.2 Collagen and GAG development

**FIGURE 2:**
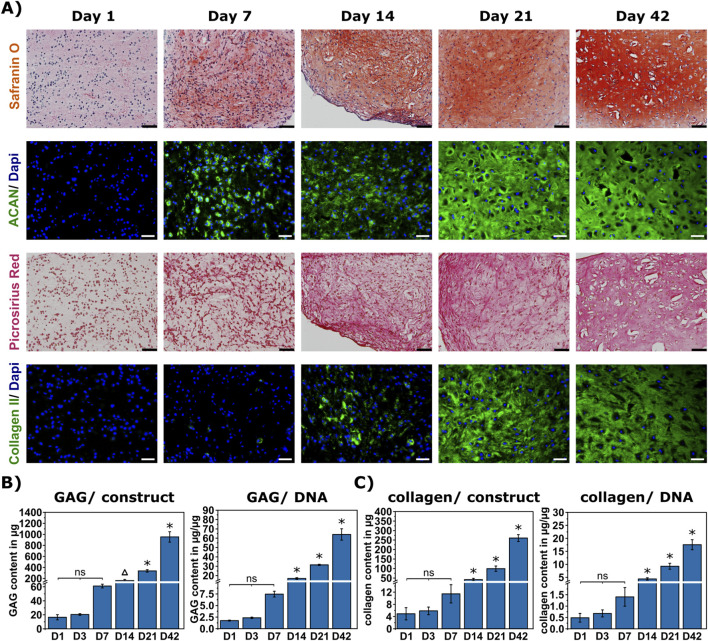
ECM development during chondrogenic differentiation over 42 days. **(A)** Histological and immunohistochemical (IHC) stainings: Safranin O staining for total GAG, picrosirius red staining for total collagen, scale bar represents 100 μm, and IHC staining of ACAN and COL II using DAPI as counterstain, scale bar represents 50 μm. **(B)** Quantification of GAG per construct (left) and normalized to the DNA (right) over time (D1/14/21/42: n = 3, D3/7: n = 4). **(C)** Quantification of collagen per construct (left) and normalized to the DNA (right) over time (D1/14/21/42: n = 3, D3/7: n = 4). Data are presented as mean ± standard deviation; statistically significant differences (p < 0.05) to all other time points are marked by *, and to all other time points except to D7 by Δ; ns = no significant difference within marked time points.

Analysis of the development of cartilaginous ECM was performed during a 42-day differentiation period at six different time points (day 1, 3, 7, 14, 21, and 42). At all time points, the constructs showed a good cell survival (Supplementary Figure S1). The production of cartilaginous ECM components was visualized with histology and immunohistochemistry at all time points (Figure 2A, not showing day 3). No visual difference was detected between day 1 and day 3. First visible changes were observed at day 7 in the safranin O staining of glycosaminoglycans (GAG) as well as the specific immunohistochemical (IHC) staining for aggrecan. Slight changes were also visible in the picrosirius red staining for collagens and specific IHC staining for collagen II, becoming more prominent at day 14. A homogeneous matrix distribution was visible for all stainings at day 21 and day 42 (Figure 2A). Collagen type I was slightly visible at day 7, with a markedly lower intensity than collagen II. Collagen X was slightly visible at day 21, becoming more prominent at day 42 (Supplementary Figure S2).

The total amount of collagen and glycosaminoglycans were also quantified at all time points using biochemical assays. An increase in GAG and, slightly less pronounced, in collagen was initially observed at day 7, followed by significant increases from day 14 onwards (Figures 2B,C). Hence, the biochemical results well represented the histological stainings.

### 3.2 Influence of the ECM development on the mechanical properties of cartilaginous constructs

**FIGURE 3:**
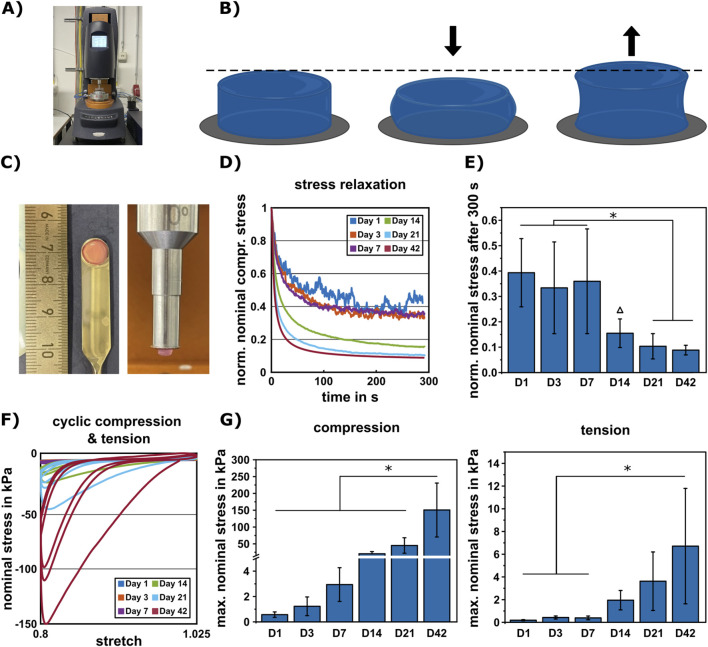
Mechanical characterization of cartilaginous constructs during 42-day period. **(A)** Testing setup using the Discovery HR-3 rheometer from TA instruments (New Castle, DE, United States). **(B)** Multimodal mechanical testing of chondrogenic constructs in compression and tension (see also Table 1 for specific testing protocol). **(C)** Exemplary images of construct size and attachment to the rheometer, prior to mechanical measurements. **(D)** Average normalized stress relaxation behavior over time. **(E)** Corresponding stress relaxation values in compression at a maximum strain of 20% after 300 s (D1: n = 4, D3: n = 6, D7: n = 5, D14/21/42: n = 7). **(F)** Cyclic loading behavior at all measured time points (single curves represented in Supplementary Figure S4). **(G)** Average maximum nominal stress during cyclic compression-tension measurement (D1/14/21: n = 5, D3: n = 7, D7: n = 4, D42: n = 6). Data are presented as mean ± standard deviation, statistically significant differences are indicated by * (p < 0.05), timepoint only significant to D1 indicated by Δ.

Large-strain multimodal mechanical testing on Discovery HR-3 rheometer in compression and tension (Figures 3A–C) was performed during the 42-day differentiation period at the same time points as the ECM analyses. An overview of the testing protocol is shown in Table 1.

#### 3.2.1 Stress relaxation behavior in compression

To quantify the viscoelastic properties of the constructs, the normalized stress relaxation behavior in compression and the corresponding nominal stresses after 300 s were analyzed. During 300 s of holding in compression, the cell-laden HA-SH hydrogels at all six time points showed a characteristic viscoelastic response, but only samples at day 21 and 42 almost reached equilibrium (Figures 3D,E). While cell-laden HA-SH hydrogels showed similar relaxation behavior within the first week of differentiation, i.e., down to 0.39, 0.33, and 0.36 normalized nominal stress in compression within 300 s of holding, samples after 2, 3, and 6 weeks of differentiation relaxed significantly faster and down to 0.15, 0.1, and 0.09, respectively (Figures 3D,E). Constructs without embedded cells that were stored in PBS for 21 days showed a stress relaxation behavior similar to constructs with cells at day 1 (Supplementary Figure S3A).

#### 3.2.2 Cyclic loading behavior in compression and tension and corresponding material parameters

To quantify the mechanical properties including stiffness, nonlinearity, hysteresis, and conditioning behavior of the constructs, the cyclic loading behavior in compression and tension at all six time points was analyzed (Figures 3F,G; single time points see Supplementary Figure S4). Significant differences in the maximum nominal stresses, the force divided by the original cross-sectional area, in compression were revealed after the total differentiation period of 42 days to all other time points, reaching a value of 151 kPa (Figure 3G; Supplementary Figure S4). Under tensile loading, significant differences were observed after 42 days to the first week of differentiation, reaching a maximum nominal stress of 7 kPa (Figure 3G; Supplementary Figure S4). Besides an increase in nominal stress, a more distinct nonlinearity and a more pronounced hysteresis (265-fold) were found with increasing time from day 1 to day 42 (Supplementary Figure S6). Furthermore, substantial preconditioning during the first loading cycle and minor conditioning effects during the subsequent cycles in compression and tension were observed at all time points (Figure 3F; Supplementary Figure S4). Changes in mechanical properties of cell-free constructs within 21 days were negligible compared to cell-containing constructs (Supplementary Figures S3B–G).

To characterize the nonlinear, time-independent response of cartilaginous constructs, the nonlinearity parameters *α*_*i*_ and the shear moduli *μ*_*i*_ of the one- and two-term Ogden model were inversely determined by fitting these models simultaneously to experimental compression and tension data (Supplementary Figure S5). Based on the coefficient of determination *R* ^2^, quantifying the goodness of the fit between model and experimental data, either the one- or two-term Ogden model was used to determine *α*_*i*_ and *μ*_*i*_. The one-term Ogden model was capable of capturing the softer mechanical properties of cell-containing constructs at early analysis time points (d1, d3, d7, *R* ^2^ = 0.90-1.00), while only the two-term Ogden model was capable of capturing the stiffer, highly nonlinear behavior of responses at later time points (d14, d21, d42, *R* ^2^ = 0.99-1.00) (Supplementary Figures S5A,B; Supplementary Figure S6C,E). An increase in the classical shear modulus (*µ*) from day 1 to day 42 up to 50.2 kPa was detected (Supplementary Figure S6C) (equaling an apparent Young’s modulus of 150.6 kPa, see Equation 9). This corresponded to a 174-fold and 69-fold increase for the unconditioned (1^st^ measurement cycle) and conditioned (3^rd^ measurement cycle) responses, respectively (Supplementary Figure S6D).

### 3.3 Correlation between cartilaginous ECM and mechanics

**FIGURE 4:**
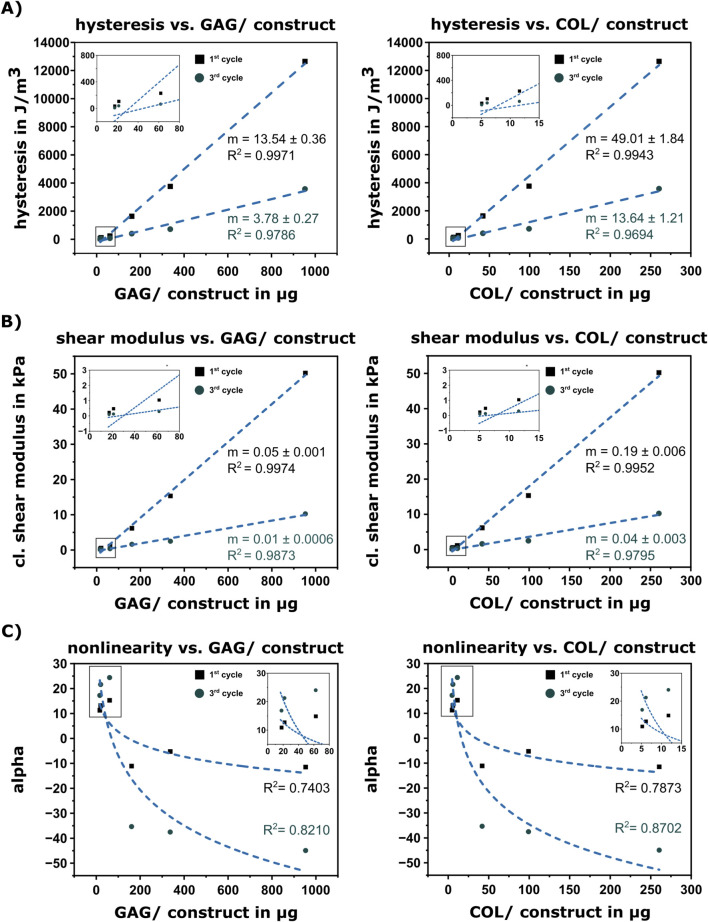
Correlation between ECM quantity and mechanical behavior. **(A)** Linear fit between hysteresis (first and third cycle of cyclic measurement) and ECM components GAG (left) and collagen (right). **(B)** Linear fit between classical shear modulus and ECM components GAG (left) and collagen (right). **(C)** Logarithmic fit between nonlinearity (alpha) and ECM components GAG (left) and collagen (right). Linear and logarithmic fits are shown for the first and third measurement cycle of cyclic loading, respectively. For clarity, insets show the data in the low-concentration range.

Statistical correlation between mechanical behavior (hysteresis, classical shear modulus, nonlinearity) and cartilaginous ECM was performed. A positive linear correlation with a *R* ^2^ >0.96 was detected between the total amount of GAG as well as COL per construct and the quantified hysteresis during the first and third measurement cycle (Figure 4A). Similarly, a positive linear correlation was detected between the total amount of GAG as well as COL per construct and the classical shear modulus, with a coefficient of determination *R* ^2^ >0.995 for the unconditioned response (1^st^ cycle) and *R* ^2^ >0.97 for the conditioned response (3^rd^ cycle) (Figure 4B). A logarithmic correlation was detected between the amount of GAG as well as COL per construct and the nonlinearity parameter α with *R* ^2^ values of 0.74 and 0.79, respectively, with an increase to *R* ^2^ >0.82 for the third measurement cycle (Figure 4C).

### 3.4 Inhibition of collagen production by prolyl 4-hydroxylase inhibitor

**FIGURE 5:**
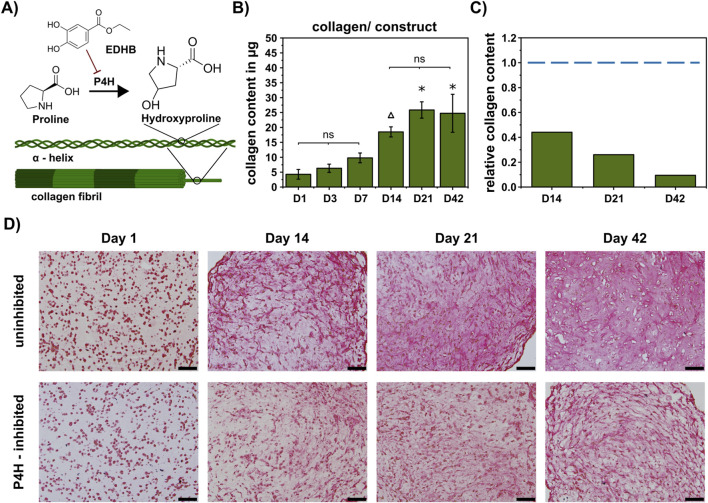
Inhibition of collagen production by the P4H inhibitor EDHB. **(A)** Scheme of inhibition mechanism. **(B)** Quantification of collagen per construct during 42-day chondrogenesis (D1/3/7/14/21/42: n = 3). Data are presented as mean ± standard deviation, statistically significant differences (p < 0.05) are marked as follows: Δ significant to D1/3, *significant to D1/3/7; ns = no significant difference within marked time points. **(C)** Relative collagen content compared to uninhibited samples (dashed blue line). **(D)** Picrosirius red staining for total collagen comparing inhibited and uninhibited samples; scale bar represents 100 µm.

**FIGURE 6:**
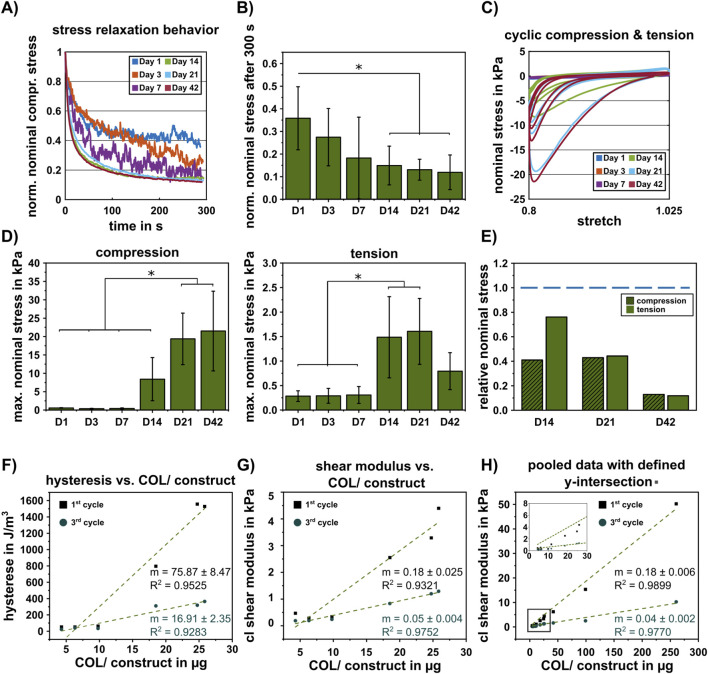
Effects of P4H inhibition on mechanical properties. **(A)** Average normalized stress relaxation behavior. **(B)** Corresponding stress relaxation values in compression at a maximum strain of 20% after 300s of inhibited constructs (D1: n = 5, D3: n = 4, D7: n = 3, D14: n = 7, D21: n = 6, D42: n = 8). Data are presented as mean ± standard deviation, statistically significant differences are indicated by * (p < 0.05). **(C)** Cyclic loading behavior at all measured time points. **(D)** Average maximum nominal stress during cyclic compression-tension measurement of inhibited constructs in compression (left) and tension (right) (D1/3/21: n = 5, D7/42: n = 6, D14: n = 8). Data are presented as mean ± standard deviation; statistically significant differences are indicated by * (p < 0.05). **(E)** Relative nominal stress in compression and tension of inhibited constructs compared to uninhibited constructs (dashed blue line). **(F)** Linear fit between quantified hysteresis and collagen in inhibited constructs. **(G)** Linear fit between classical shear modulus and collagen in inhibited constructs. Linear fits are shown for the first and third measurement cycle of cyclic loading, respectively. **(H)** Pooled dataset (uninhibited and inhibited constructs), linear fit between classical shear modulus and collagen. For clarity, inset shows the data in the low-concentration range.

To further investigate the relationship between ECM components and mechanical properties, we inhibited the prolyl 4-hydroxylase (P4H) using ethyl 3,4-dihydroxybenzoate (EDHB). The P4H hydroxylates the amino acid proline to hydroxyproline, which is a major amino acid in collagen (Figure 5A). By P4H inhibition, we aimed to impair collagen synthesis (Figure 5) and subsequently analyzed the effect on mechanical properties (Figure 6). Under P4H inhibition, an increase of total collagen could be detected after 14 days of chondrogenic differentiation, however, the amount of collagen was distinctly lower than in constructs cultured without P4H inhibition (Figure 5B, compare Figure 2C). The relative collagen content decreased to 0.44 compared to the uninhibited constructs after 14 days and continued to decrease down to 0.09 after 42 days (Figure 5C, compare Figure 2C). Collagen content was additionally analyzed using picrosirius red staining, showing a less intense staining pattern compared to uninhibited constructs (Figure 5D).

Stress relaxation behavior showed similar results compared to the uninhibited samples, decreasing to 0.35 normalized nominal stress at day 1, with a continuous decrease down to 0.11 after 42 days (Figures 6A,B, compare Figures 3D,E). The inhibited constructs showed a distinct nonlinear behavior and pronounced hysteresis during the entire cultivation time from day 1 to day 42 (Figure 6C; Supplementary Figure S8). Significant increase in the maximum nominal stress under compressive loading was detected after 21 days, with no significant difference between 21 and 42 days. Significant differences under tensile loading were detected after 14 days, with no significant differences to 21 or 42 days (Figure 6D). The relative nominal stress compared to the uninhibited samples decreased distinctly between 14 and 42 days of differentiation, resulting in a relative nominal stress of 0.1 for compression and tension at day 42 (Figure 6E).

Similar to the uninhibited constructs, the mechanical responses of inhibited constructs at early analysis time points (d1, d3, d7, *R* ^2^ = 0.94-0.99) could be captured by the one-term Ogden model, while the two-term Ogden model for later time points (d14, d21, d42, *R* ^2^ = 0.94-1.00) was needed to capture the increasing nonlinear behavior (Supplementary Figures S7, S8). A strong positive correlation could again be detected between the total amount of collagen per construct and the quantified hysteresis with a *R* ^2^ = 0.92-0.95, as well as between the total amount of collagen per construct and the classical shear modulus with a *R* ^2^ = 0.93-0.97 (Figures 6F,G).

### 3.5 ECM-based prediction of mechanical properties

Based on the correlations in Figures 4B, 6G, we introduce the classical shear modulus *µ* of chondrogenic constructs as a linear function of the amount of collagen in µg (COL) with the slope *m* (COL) and the y-intercept c (Equation 10). Based on the classical shear modulus of the HA-SH hydrogel samples without cells (Supplementary Figure S3E), we set *c* = 0.247. To accurately calibrate the parameter m (COL), we used the pooled datasets for the unconditioned (1^st^ cycle) samples, namely, both uninhibited (Supplementary Figure S6C) and inhibited samples (Supplementary Figure S8C), resulting in the following mechanical prediction function

$$µ\left(COL\right)=0.18\times COL+0.247$$ (10)

The prediction function captures the classical shear modulus of unconditioned chondrogenic constructs dependent on the amount of collagen (Figure 6H, *R* ^2^ = 0.99).

## 4 Discussion

Achieving the complex mechanical properties of native cartilage through regenerative tissue engineering strategies still represents a great challenge (Petitjean et al., 2023; Welsh and Sikder, 2025). Various 3D bioprinting approaches are being investigated to improve the quality of engineered articular cartilage, however, most studies do not characterize the mechanical properties under large deformations capturing physiological conditions such as standing or walking. Here, using a stand-alone bioink approach, we determined the ECM development of engineered constructs over time and performed a time-matched multimodal analysis of mechanical characteristics. We demonstrated strong correlations between ECM content and specific mechanical properties of the constructs.

### 4.1 Bioprinting and chondrogenesis

In our study, we could demonstrate reproducible printability comparable to the originally established bioink formulation (Hauptstein et al., 2022), despite a different molecular size of the HA-SH. Printability was further proven to be stable without and with embedded cells, which confirmed previous results by a study using a GelMA-based bioink indicating only slight changes in printability up to 40*10^6^ cells per ml bioink (Gillispie et al., 2020). The mere cell embedding also did not cause any changes in the microstructure of the bioink. However, it decreased the pore size during 21 days of differentiation, as compared to day 1 or bioinks without embedded cells, which was most likely due to the synthesis of ECM components. While aggrecan was detectable after 7 days via IHC and GAG showed significant changes in the biochemical quantification after 14 days, both collagen II in IHC and significant quantified amounts of total collagen were detected after 14 days. Distinct ECM accumulation continued over the whole culture period of 6 weeks. After 42 days, also a prominent deposition of collagen X was observed, which is a marker of chondrocyte hypertrophy. While many studies have investigated how to potentially prevent hypertrophy (Gawlitta et al., 2012; Chen et al., 2015; Wang et al., 2017), it still remains a challenge in the chondrogenic differentiation of MSC. Despite the fact that many studies have shown a variance in ECM production depending on the cell type and donor age (Neybecker et al., 2020; Bagge et al., 2022; Veenendaal et al., 2025), in general, the observed ECM production was in agreement with previous studies investigating the chondrogenic differentiation of MSC in cell aggregates (Sorrell et al., 2018; Vakhrushev et al., 2024) as well as in hydrogel systems (Levato et al., 2017; Lan et al., 2024).

### 4.2 Mechanical changes and correlation with ECM components

During the 42-day differentiation period, all analyzed samples showed a viscoelastic mechanical behavior during cyclic large-strain compression-tension and stress relaxation experiments. The increasing ECM development markedly changed the overall multimodal mechanical response of the constructs. In some biofabrication approaches using fiber-reinforced bioinks a considerable initial construct stiffness has been reported due to the structure itself (Spauwen et al., 2025), however, the fiber design potentially impairs development of cartilaginous tissue with homogeneous ECM over time. In contrast, constructs made from stand-alone bioinks typically exhibit a mechanical resistance that is distinctly lower than that of constructs with support structure (Levato et al., 2017; Lan et al., 2024), however, they permit homogeneous tissue development, and the ECM produced during the cultivation period can result in distinctly improving mechanical properties over time. In our stand-alone bioink, the increase of ECM did not only impact the overall stiffness, but it also changed the stress relaxation behavior. While it is known from the literature that the stress relaxation behavior of hydrogels can impact the ECM distribution within the hydrogel (Lee et al., 2017), we could demonstrate a distinct change in stress relaxation caused by the ECM production over time. Additionally, the hysteresis in cyclic compression-tension experiments increased during the differentiation period, indicating an increased viscoelastic response after 42 days. Interestingly, during repeated cyclic loading, the hysteresis decreased between the first and third cycle. This might be associated with a rearrangement of the collagen fibers, the water binding properties of GAG (Petitjean et al., 2023), as well as related porous effects due to free-flowing fluid within the material. These phenomena might have also led to the softening tissue response after the first cyclic loading. Furthermore, the increasing amount of ECM over time also resulted in a change in nonlinearity, i.e., a more pronounced nonlinear behavior of the constructs in compression at later time points.

Taken together, using our multimodal analysis we observed several mechanical characteristics for our cartilaginous constructs also similarly reported for native articular cartilage. Cyclic loading behavior with a classical shear modulus of 50.2 kPa after 42 days and a stress relaxation behavior up to 91% within 300 s in compression of the constructs were comparable to articular cartilage with a classical shear modulus of 60.4 kPa and a stress relaxation behavior between 79% and 84% within 300 s in compression (Faber et al., 2025a). Furthermore, the constructs closely mimicked the highly nonlinear, hysteretic behavior of articular cartilage with substantial conditioning during cyclic loading (Little et al., 2011; Weizel et al., 2020; Faber et al., 2025a). With regard to the maximal nominal stress in compression and tension, the constructs reached values of 150 kPa and 6 kPa, respectively, that were still distinctly below those of articular cartilage (600–800 kPa and 50–200 kPa) (Faber et al., 2025a). Nevertheless, associated with progressing ECM development, clear increases in nominal stress over time were observed for the engineered constructs. Also, it must be considered that our constructs have been cultured under static conditions. Several previous studies have reported a possible increase of cell migration and ECM production under the influence of dynamic culture conditions (Vaca-Gonzalez et al., 2019; Gilbert et al., 2021; Goldberg-Bockhorn et al., 2022). Whether a dynamic culture has a positive impact on the development of the constructs presented here or affects the degradation rate of the stand-alone bioink may be investigated in future studies. Overall, while most biofabrication approaches lack a detailed mechanical analysis and often focus on single values, such as the Young’s modulus, our study emphasizes the importance of a more extensive mechanical characterization, by showing multiple alterations in the mechanical development of chondrogenic constructs.

In this study, we were additionally able to demonstrate a very strong correlation between the amount of GAG as well as the amount of collagen and the classical shear modulus quantifying the stiffness. The correlation also held for constructs with an inhibited collagen synthesis, causing a decrease of collagen amount and stiffness, which confirmed the robustness of the observed correlations. It is known that GAG has a stiffening effect under compressive loading, due to the fact that the negatively charged molecules bind water, building an osmotic pressure within the construct (Nimeskern et al., 2016). Collagen fibers provide tensile strength, due to their fibrillar network; fibers buckle in compression but provide resistance in tension (Sophia Fox et al., 2009). In our constructs, we could demonstrate the impact of collagen on tensile stresses as well as compressive stresses: the depletion of collagen also influenced the stress response in compression. It has to be noted that for a correlation between the amount of ECM components and the mechanical properties, one requirement is a hydrogel material that enables unimpaired diffusion of synthesized ECM components throughout the construct. It has been shown in non-printed (Erickson et al., 2009; Bian et al., 2013) and bioprinted constructs (Hauptstein et al., 2020; Hauptstein et al., 2022; Lan et al., 2024; Perry et al., 2025) that a too high polymer concentration or crosslinking density resulted in impaired diffusion leading to an only pericellular ECM distribution, which in turn yielded decisively lower construct stiffness than in constructs with unimpaired diffusion and a resulting homogeneous ECM distribution (despite comparable ECM amounts). Here, we utilized our previously developed bioink platform (Hauptstein et al., 2022), confirming a homogeneous ECM distribution for the employed formulation. While in the current study, the focus was on the relationship between the mere amount of collagen and the mechanical properties, future work may also investigate into the influence of collagen organization. Furthermore, besides the direct effects of the collagen molecules, potential reasons for the collagen impact on the mechanical properties of the constructs may also include the influence of other matrix components, such as MATN3 and COMP, which are known to interact with collagen and maintain the structural integrity of cartilage (Budde et al., 2005; Maly et al., 2021). While we could demonstrate a positive correlation between collagen/GAG content and mechanical stiffness consistently during a 42-day period, the influence of subsequent changes of matrix composition beyond that period and the potential influence of smaller linker proteins like MATN3 and COMP are to be further analyzed in future studies.

To the best of our knowledge, this is the first detailed correlation study between extracellular matrix and mechanical behavior over time in bioprintable constructs. Most biofabrication approaches perform mechanical analysis of the material itself (Faber et al., 2025b) or an end- or two-time-point analysis of the constructs and, moreover, focus on single mechanical parameters (Levato et al., 2017; Hauptstein et al., 2022; Lan et al., 2024; Puiggali-Jou et al., 2025; Spauwen et al., 2025). In some non-printed tissue engineering approaches, multimodal mechanical analysis has been performed previously and related to construct maturation over time. In this context, early studies in alginate-based hydrogels have investigated the influence of cell density and dynamic loading, finding that in general a higher cell density led to an earlier increase in matrix quantity, concomitantly causing significant changes in mechanical behavior (Mauck et al., 2002; Wan et al., 2011). A study on hyaluronic acid-based hydrogels has shown the impact of polymer concentration on ECM content and distribution, the latter distinctly influencing multiple mechanical parameters under compression and tension over time (Erickson et al., 2009). A study using collagen scaffolds has shown changes in multiple mechanical properties corresponding with the amount and distribution of GAG within 7 weeks of cultivation (Middendorf et al., 2017). A recent approach has evaluated a new non-destructive imaging-based analysis technique in comparison to established compression tests to investigate tissue maturation, confirming the correlation between matrix and mechanical properties in self-assembled constructs (Haudenschild et al., 2022). In our current study, we investigated tissue maturation and mechanical development in biofabricated constructs and could corroborate earlier findings of the aforementioned studies regarding a time-matched development of ECM and viscoelastic properties.

In summary, our comprehensive post-fabrication analysis demonstrated that the ECM development markedly influenced the overall multimodal mechanical response over 6 weeks. Clear changes in nonlinear and stress relaxation behavior and increases in hysteresis and the classical shear modulus were shown, and distinct similarities between the mechanical behavior of biofabricated constructs and native articular cartilage were observed (Weizel et al., 2020; Faber et al., 2025a). Our data demonstrates a positive and robust correlation between the classical shear modulus and the ECM components during chondrogenic differentiation of MSC in the engineered constructs. Based on this correlation, we have proposed a prediction function that may aid in the design of biomaterials for cartilage regeneration (Ahmadi Soufivand et al., 2025). Different bioink systems enabling homogeneous matrix distribution can now be utilized to investigate the broader application of the prediction function. Overall, our findings contribute to the understanding of tissue maturation in engineered cartilaginous constructs.

## Data Availability

The original contributions presented in the study are included in the article/Supplementary Material, further inquiries can be directed to the corresponding authors.
