# Supplementary material for: Tissue maturation and development of mechanical properties in hyaluronic acid bioink-based cartilaginous constructs
Source: Front Bioeng Biotechnol. 2026 Apr 8;14:1749259. doi: 10.3389/fbioe.2026.1749259 (PMC13099909; doi:10.3389/fbioe.2026.1749259)
Supplement: Supplementary file 1 [file DataSheet1.pdf]

frontiers

Supplementary Material

Tissue maturation and development of mechanical properties in hyaluronic acid bioink-based cartilaginous constructs

Paula Büttner[1†], Jessica Faber [2†], Jörg Teßmar[3], Philipp Stahlhut[3], Silvia Budday[2*], Torsten Blunk[1*]

[1]Department of Trauma, Hand, Plastic and Reconstructive Surgery, University Hospital Würzburg, Würzburg, Germany

[2] Institute of Continuum and Biomechanics, Friedrich-Alexander-Universität Erlangen-Nürnberg, Fürth, Germany

[3]Department of Functional Materials in Medicine and Dentistry and Bavarian Polymer Institute, University Hospital Würzburg, Würzburg, Germany

*** Correspondence:**
Torsten Blunk, blunk_t@ukw.de
Silvia Budday, silvia.budday@fau.de

† These authors contributed equally to this work and share first authorship

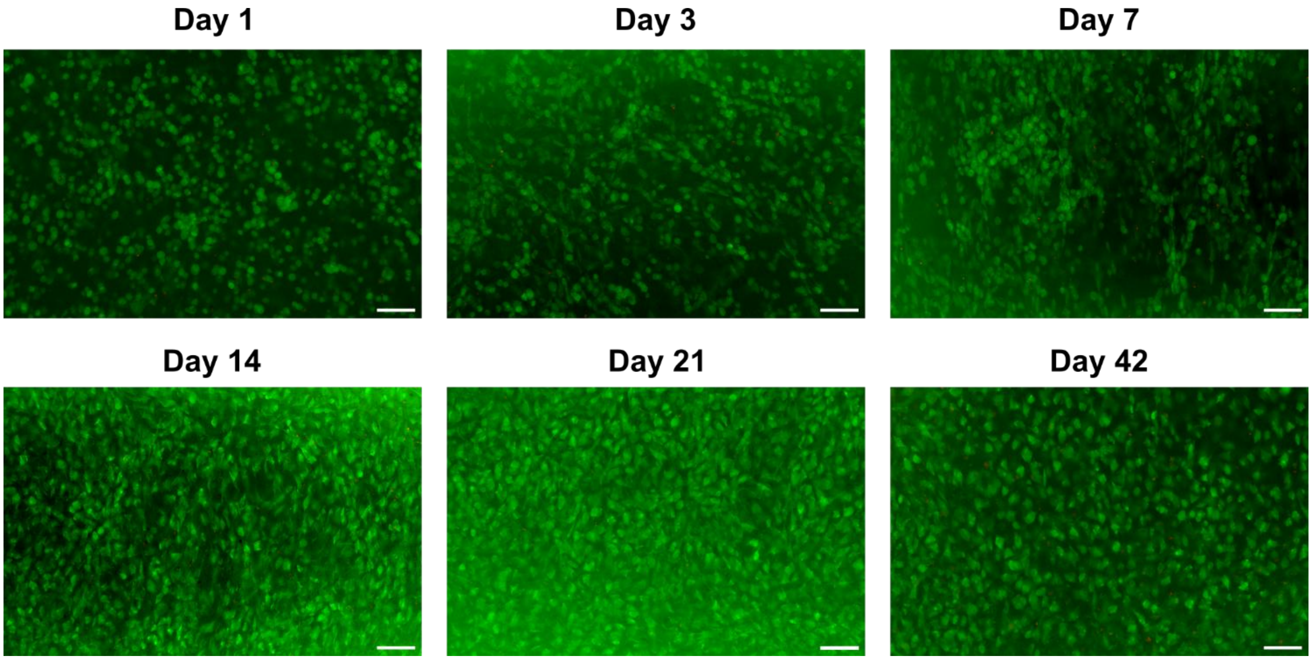

Figure S1: Live / dead staining of embedded cells in constructs. After 1/3/7/14/21/42 days, living cells were stained using calcein-AM and dead cells were stained using EthD-III, scale bar represents 100 µm.

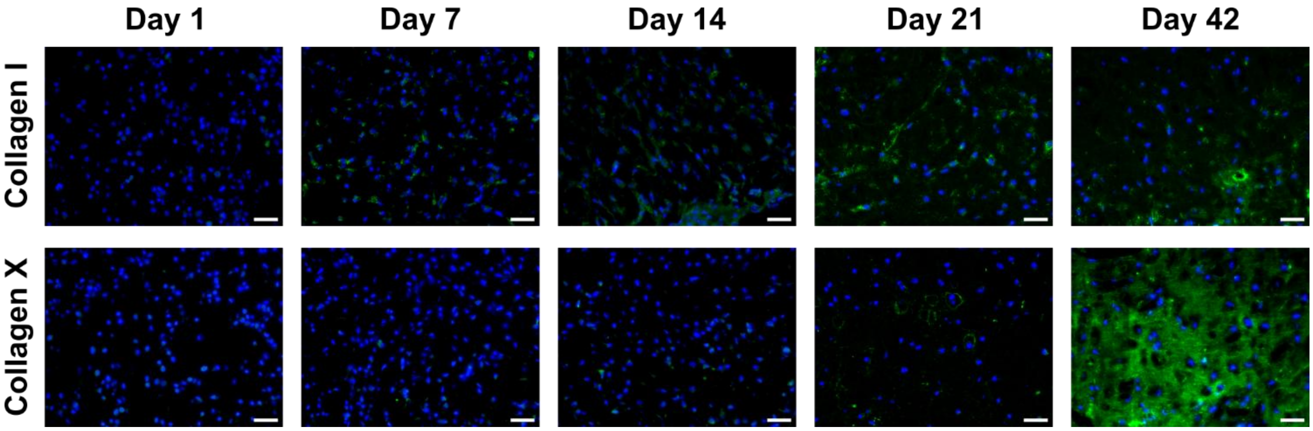

Figure S2: Immunohistochemical staining of collagen type I and collagen type X in constructs over time. DAPI was used as counterstain for nuclei; scale bar represents 50 µm.

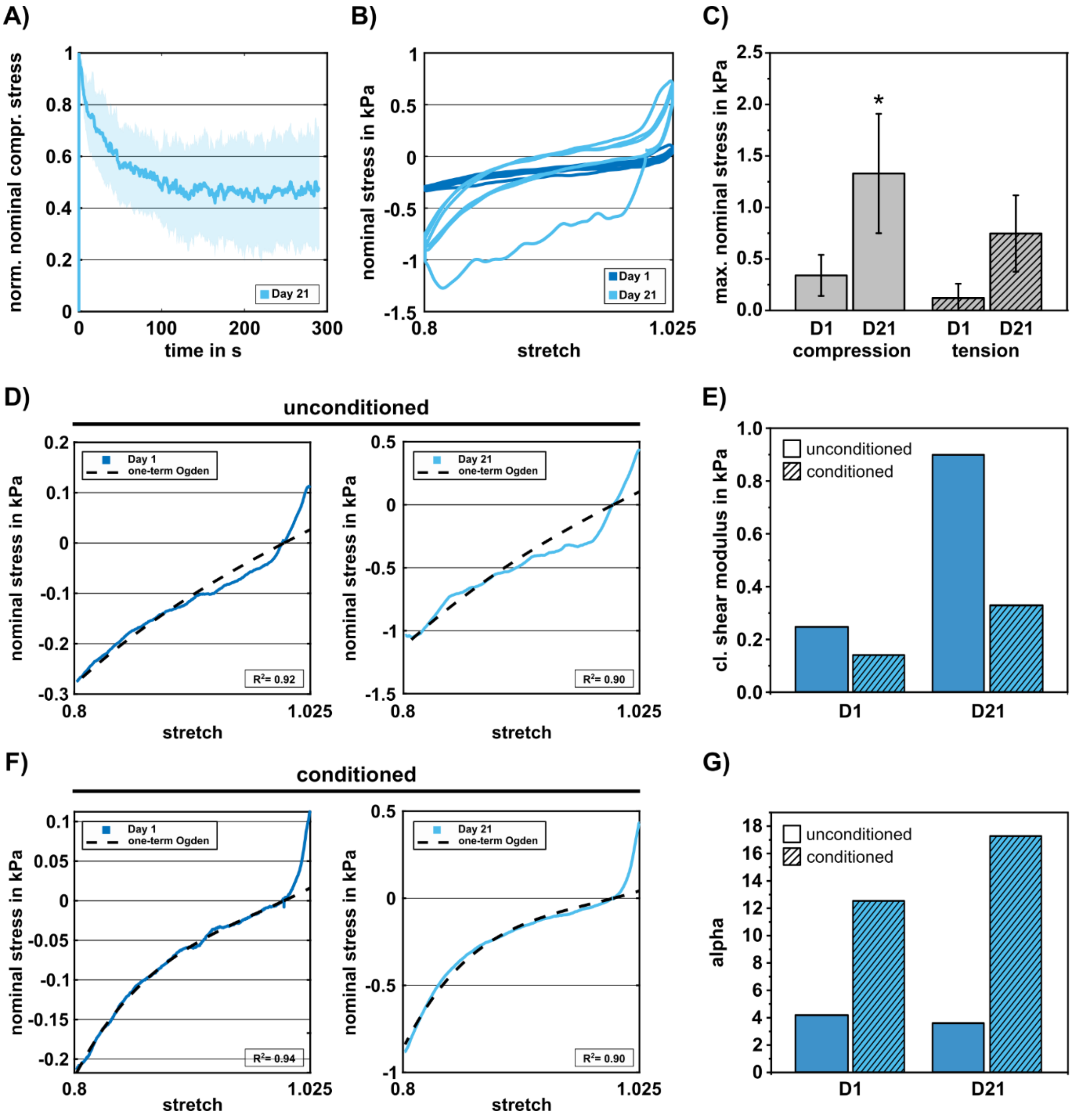

Figure S3: Mechanical analysis of HA-based constructs without cells. (A) Stress relaxation behavior of empty constructs after 21 days. (The stress relaxation behavior of ultrasoft HA-based constructs at D1 is clearly unphysical and is therefore not displayed as a result. This is strongly attributed to the low forces recorded during these tests, which reached the lower axial force limit of the machine.). (B) Cyclic loading behavior at D1 and D21 in compression and tension, displaying all three measurement cycles. (C) Average maximum nominal stress during cyclic compression-tension measurement (1st cycle) (n=5). Data are presented as mean ± standard deviation, statistically significant difference is indicated by * ($p< 0.05$). (D) One-term Ogden model calibrated with the average experimental data of D1 (left) and D21(right) of first measurement (unconditioned) cycle in compression and tension; (E) corresponding classical shear modulus of empty constructs at D1 and D21. (F) One-term Ogden model calibrated with the average experimental data of D1 (left) and D21(right) of third measurement (conditioned) cycle in compression and tension; (G) corresponding nonlinearity parameter ($\alpha 1$) of empty constructs at D1 and D21.

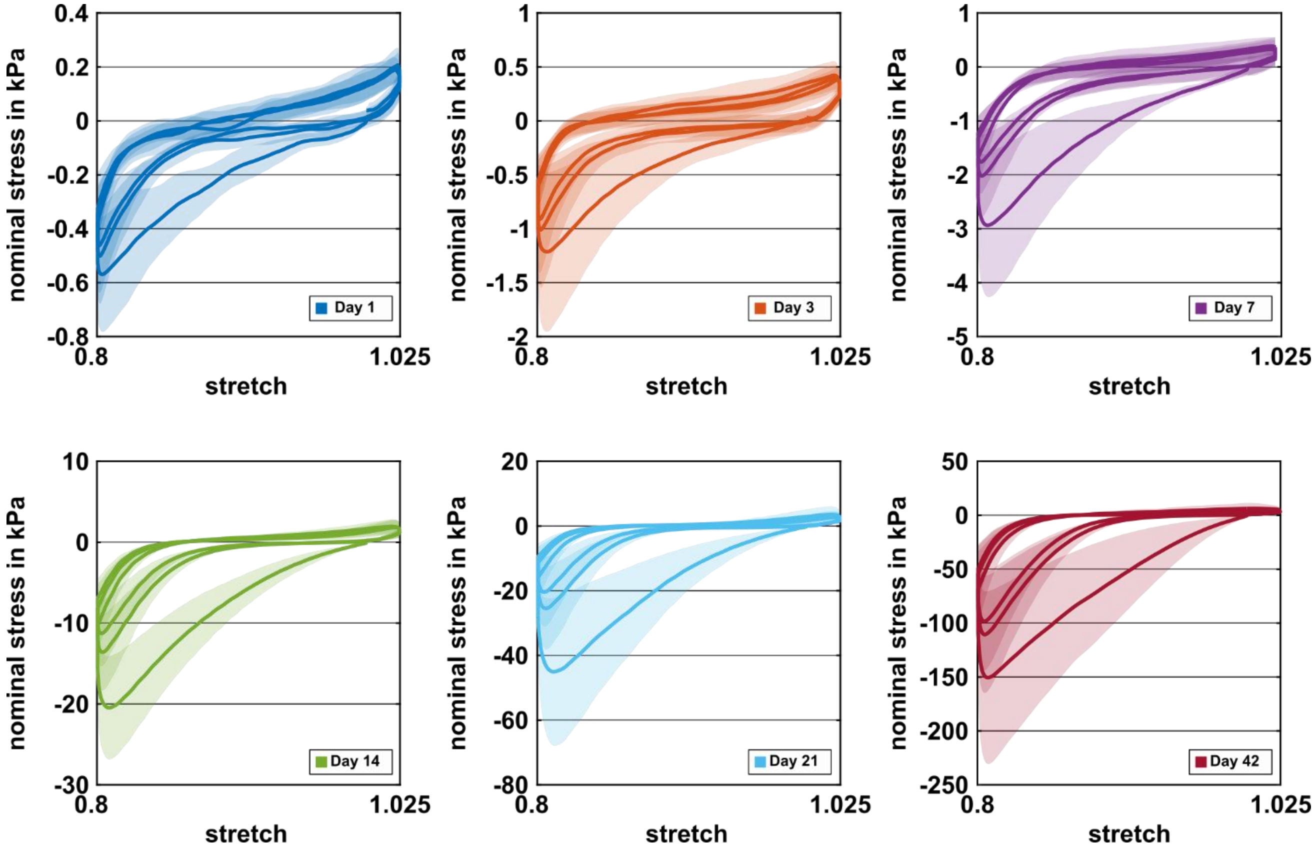

Figure S4: Cyclic loading behavior, measurement curves at single time points. Cyclic measurement (all three cycles) in compression and tension of HA-based constructs with embedded cells after 1/3/7/14/21/42 days.

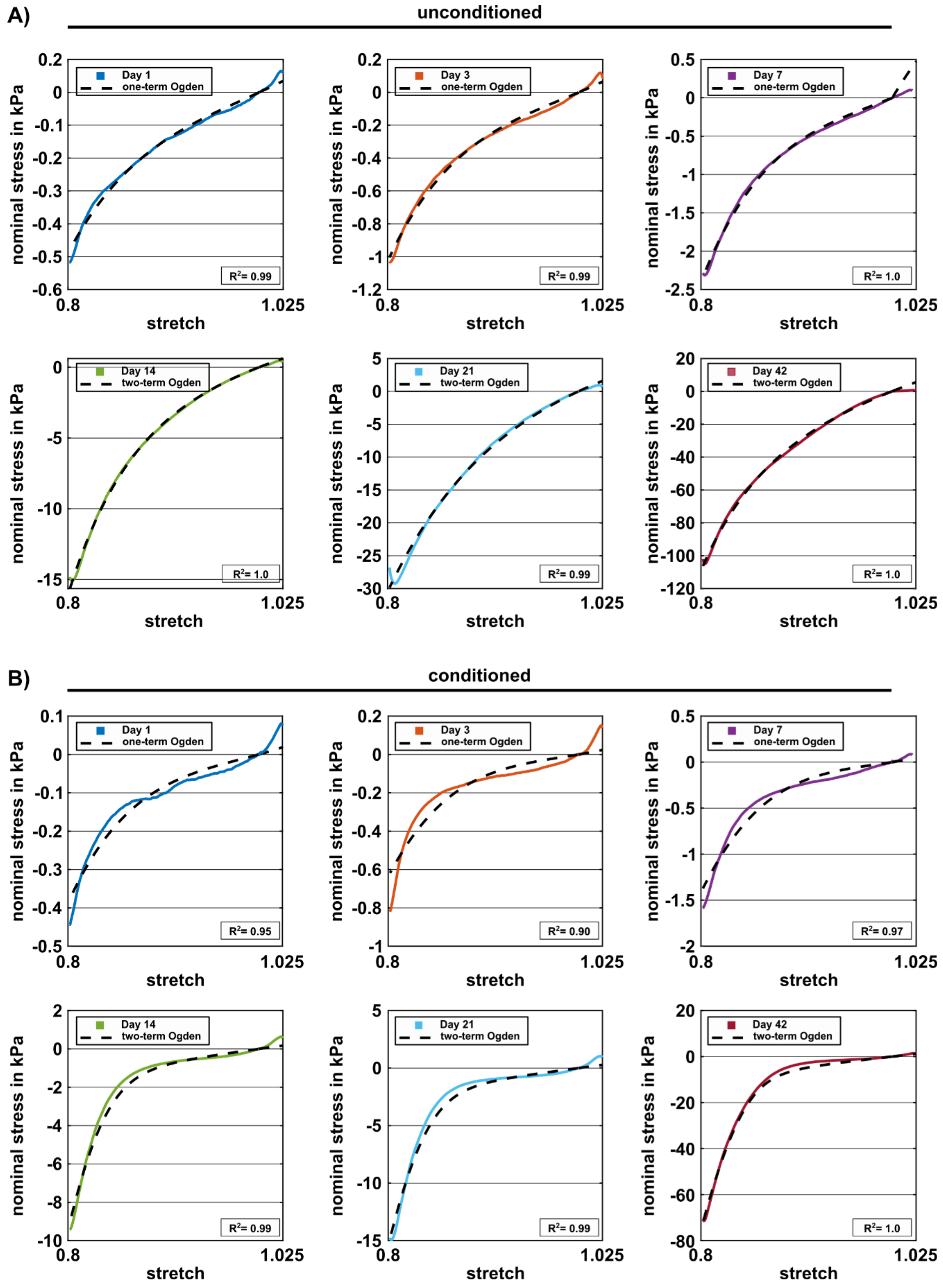

Figure S5: One- and two-term Ogden model calibrated with the average experimental data of D1, D3, D7, D14, D21 and D42 under multiple loading modes in compression and tension. (A) First measurement cycle (unconditioned); (B) third measurement cycle (conditioned).

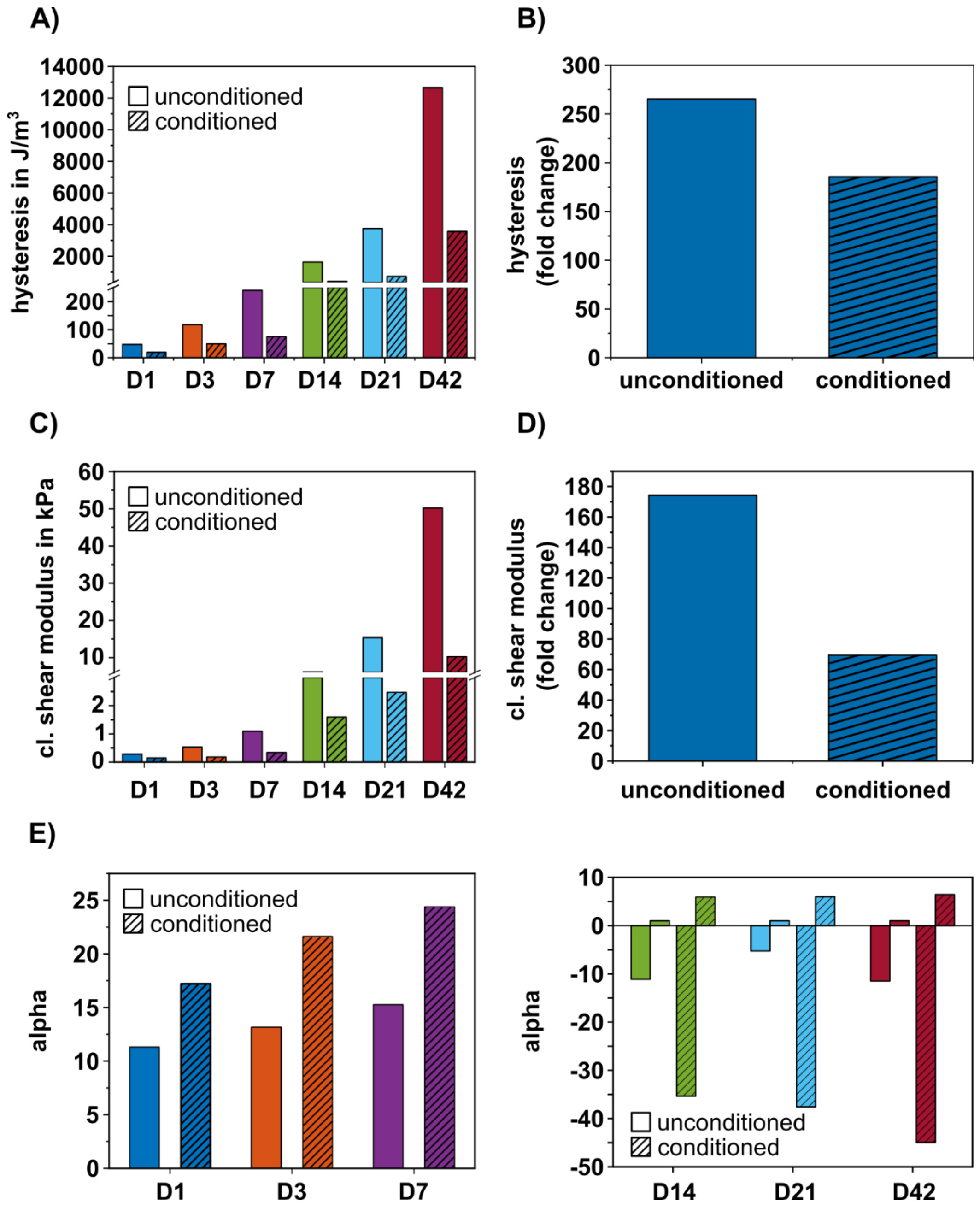

Figure S6: Hyperelastic parameters of uninhibited samples. (A) Corresponding hysteresis for the first (unconditioned) and third (conditioned) cycle of averaged cyclic compression-tension curves (D1/14/21: n=5, D3: n=7, D7: n=4, D42: n=6). (B) Fold change of hysteresis between D1 and D42. (C) Corresponding classical shear moduli μ for D1, D3, D7, D14, D21, and D42 for the first (unconditioned) and third (conditioned) cycle. (D) Fold change of classical shear modulus between D1 and D42. (E) Nonlinearity parameter ($\alpha 1$) for the modified one-term and two-term Ogden model fitted to the first (unconditioned) and third (conditioned) cycle of the averaged cyclic compression-tension curves of D1, D3, and D7 (left) and D14, D21 and D42 (right).

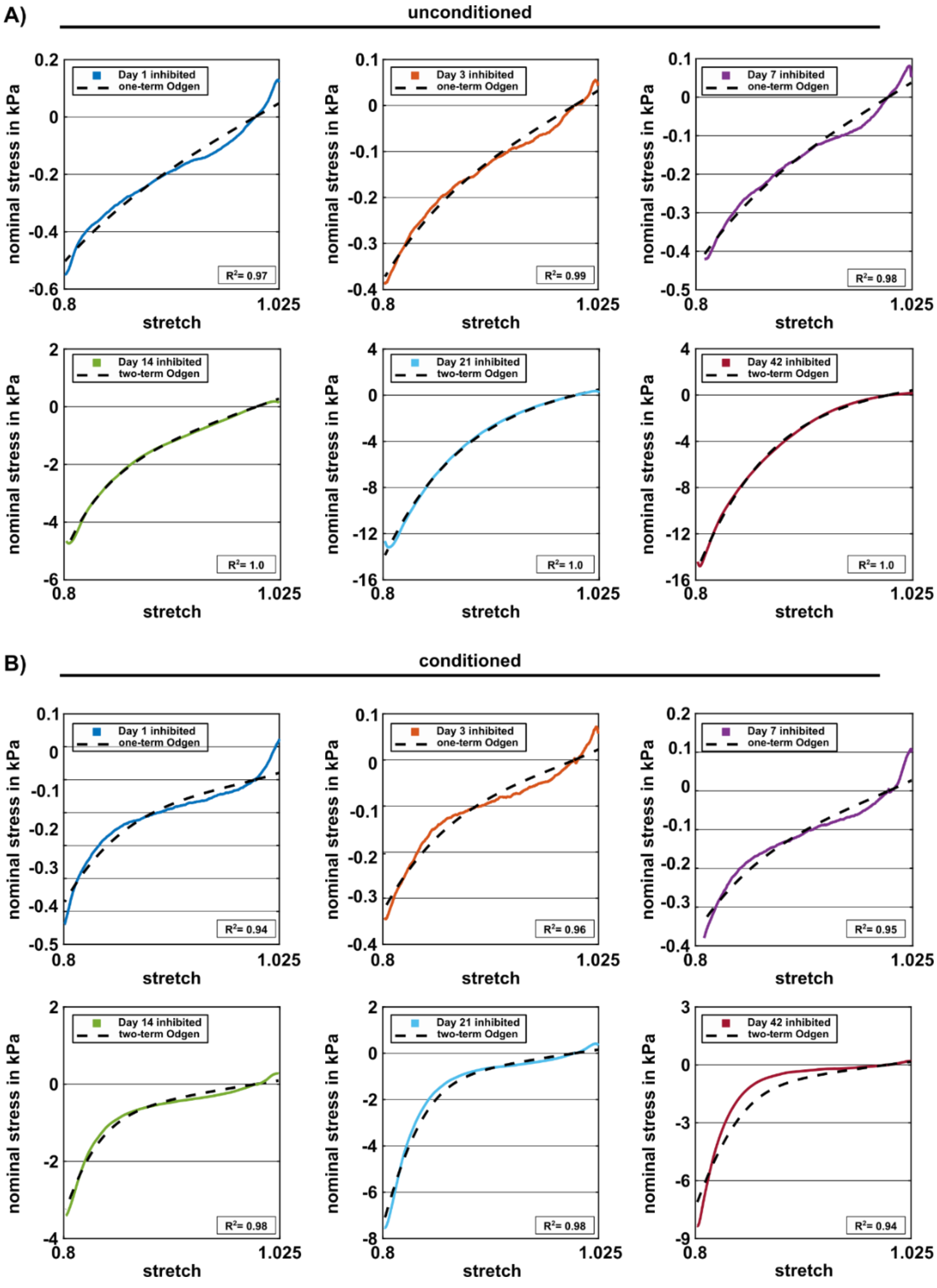

Figure S7: One-term and two-term Ogden model of P4H-inhibited samples, calibrated with the average experimental data of D1, D3, D7, D14, D21 and D42 under multiple loading modes in compression and tension. (A) First measurement cycle (unconditioned); (B) third measurement cycle (conditioned).

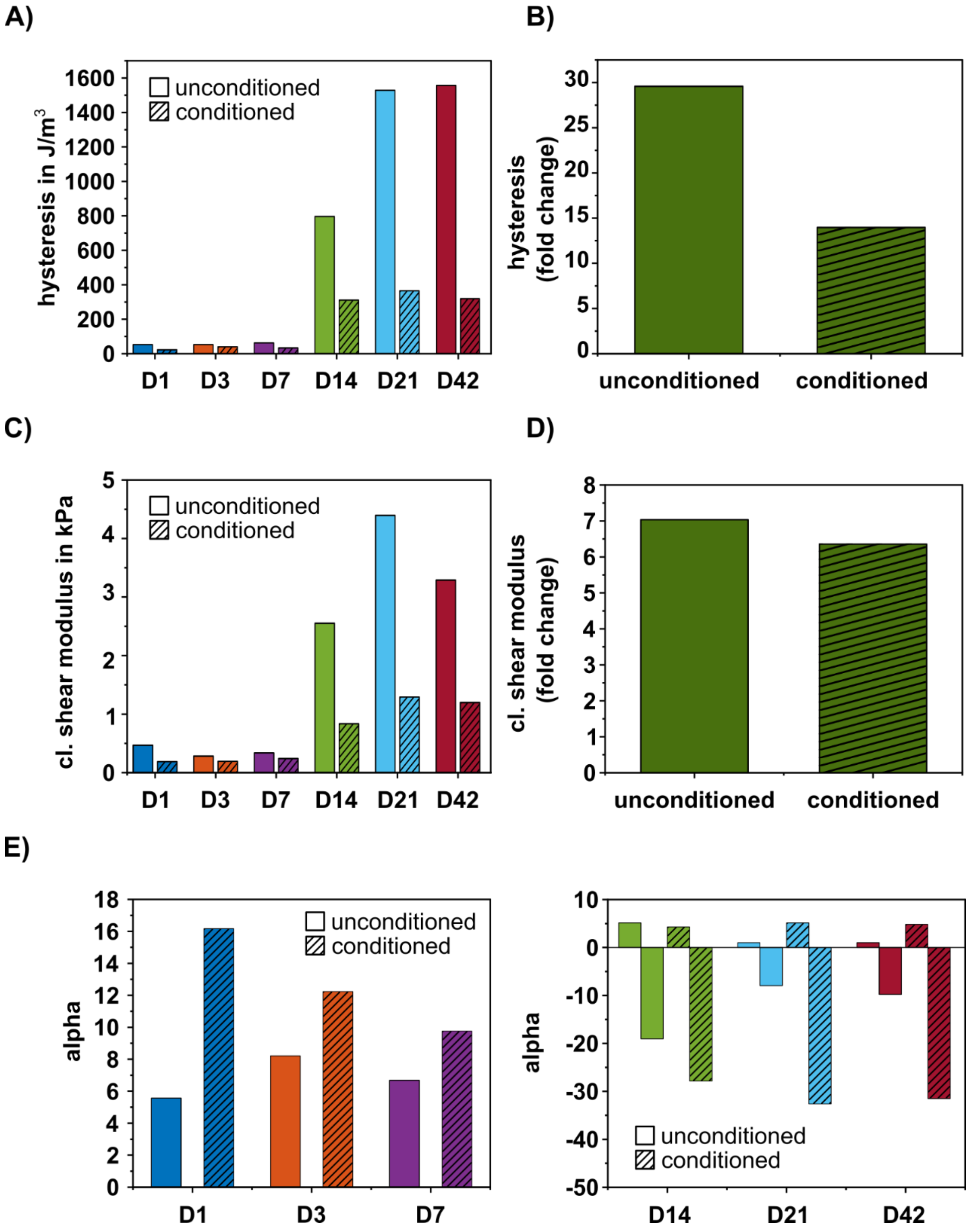

Figure S8: Hyperelastic parameters of P4H-inhibited samples. (A) Corresponding hysteresis for the first (unconditioned) and third (conditioned) cycle of averaged cyclic compression-tension curves (D1/3/21: n=5, D7/42: n=6, D14: n=8). (B) Fold change of hysteresis in inhibited samples between D1 and D42. (C) Corresponding classical shear moduli μ for D1, D3, D7, D14, D21, and D42 for the first (unconditioned) and third (conditioned) cycle. (D) Fold change of classical shear modulus of inhibited samples between D1 and D42. (E) Nonlinearity parameter ($\alpha 1$) for the modified one-term and two-term Ogden model fitted to the first (unconditioned) and third (conditioned) cycle of the averaged cyclic compression-tension curves of D1, D3, and D7 (left) and D14, D21 and D42 (right).
